# Sclerosing Fibroadenoma With Atypical Ductal Hyperplasia Mimicking Invasive Carcinoma: A Case Report With Diagnostic Pitfall

**DOI:** 10.7759/cureus.41791

**Published:** 2023-07-12

**Authors:** Shin-Ichi Murata, Hideto Iguchi, Mari Kawaji

**Affiliations:** 1 Department of Human Pathology, Wakayama Medical University, Wakayama, JPN; 2 Department of Thoracic and Cardiovascular Surgery, Wakayama Medical University, Wakayama, JPN

**Keywords:** systemic scleroderma, diagnostic pitfall, atypical ductal hyperplasia, sclerosing fibroadenoma, breast

## Abstract

Fibroadenoma (FA) of the breast is a benign fibroepithelial lesion rarely showing atypical epithelial overgrowth. We present the case of a 50-year-old Japanese woman with sclerotic FA with atypical ductal hyperplasia (ADH)/ductal carcinoma in situ (DCIS). A small mass was detected during clinical examination in the upper lateral area of the left breast. Hematoxylin and eosin stain section of a breast needle core biopsy specimen showed trabecular growth of atypical epithelial cells without distinct myoepithelial lining in the sclerotic stroma. Initial pathological diagnosis of the biopsy specimen was invasive carcinoma of no special type. The surgical specimens included a well-bordered nodular lesion with similar histological findings to that of the biopsy specimen, but, the myoepithelial lining was highlighted by cytokeratin 5 (CK5) immunohistochemistry. The tumor cells were diffusely ER-positive and completely negative for CK5 in immunohistochemical staining. Final diagnosis based on the results of immunohistochemical staining and consultation between two breast pathology specialists was the lesion as sclerosing FA with ADH/DCIS. Awareness of the unique histological subtype of FA is important to avoid pathological misdiagnosis and clinical overtreatment.

## Introduction

Fibroadenoma (FA) of the breast is a well-bordered nodular tumor of fibroepithelial lesion composed of both epithelial and stromal cell growths [1,2]. Principal histological findings of FA are intracanalicular and pericanalicular growth patterns. The intracanalicular pattern shows a compressed slit-like canalicular structure by proliferating stroma cells. The pericanalicular pattern shows open lumens of canalicular structure surrounded by expanded stroma with proliferating stroma cells. FA also has histological variations with over-growth or less-growth of epithelial and/or stromal cell components, and can be classified into complex, organized, cellular, myxoid, sclerotic, and juvenile subtypes [2].

Here, we present a case of sclerotic FA with atypical ductal hyperplasia (ADH)/ductal carcinoma in situ (DCIS) mimicking invasive carcinoma of no special type (NOS).

## Case presentation

**Figure 1:**
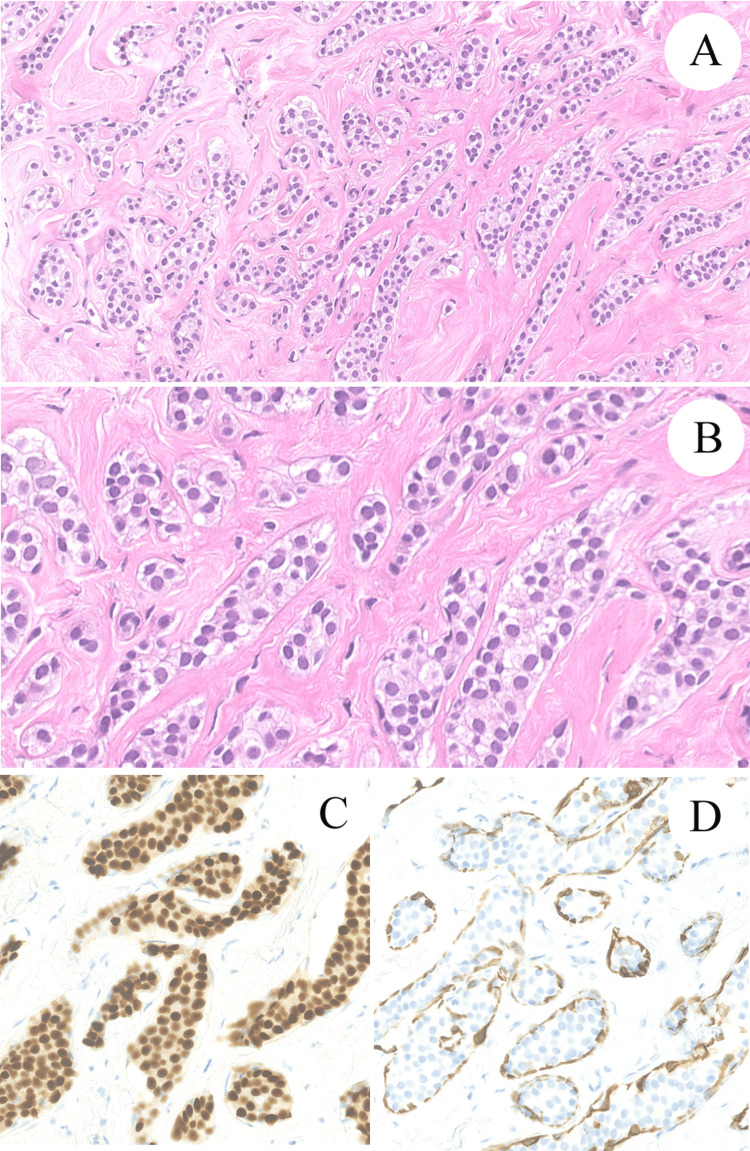
Representative histological and immunohistochemical findings of the biopsy specimen. (A and B) The tumor showed trabecular growth of mildly atypical and monotonous epithelial cells with sclerotic hyalinized stroma. (H&E, original magnification; x40 in A, x100 in B). (C) The tumor cells show diffusely positive reactivity with antibodies to ER (original magnification; x100). (D) The tumor cells are completely negative for CK5. In addition, CK5 highlighted myoepithelial lining of all tumor cell nests (original magnification; x100).

**Figure 2:**
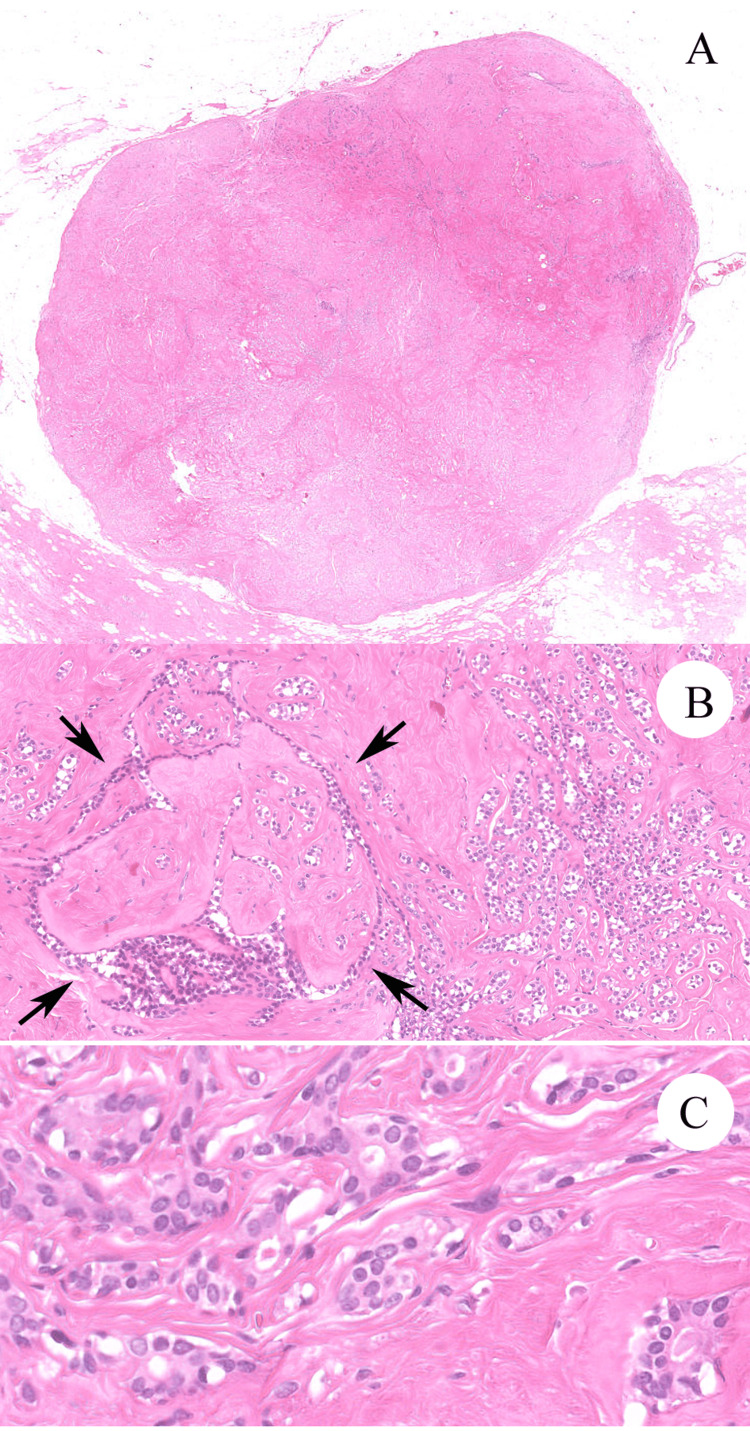
Representative histological findings of surgical specimens. (A) The surgical specimen includes a well-bordered nodular lesion (H&E, original magnification; x1). (B) Main part of the tumor shows a trabecular structure with sclerotic stroma, which is similar histological findings to those of the biopsy specimen (Figure 1a, 1b). Intracanalicular structure is only partially found (left area of the image, the area enclosed by the arrows) (H&E, original magnification; x40). (C) The tumor showed trabecular growth of mildly atypical and monotonous epithelial cells with hypocellular sclerotic stroma (H&E, original magnification; x100).

A 50-year-old Japanese woman was referred from another hospital with a mass formation of left breast. The patient had systemic scleroderma. A small well-bordered nodular lesion in the left upper lateral mammary region (the 2 o'clock position) was shown on ultrasound and computed tomography images. The clinical preoperative diagnosis was FA or invasive ductal carcinoma. A breast needle core biopsy was performed for pathological diagnosis. Hematoxylin and eosin-stained (H&E) section of the biopsy specimen showed trabecular growth of mildly atypical epithelial cells with hypocellular sclerotic stroma (Figure 1a, 1b). Myoepithelial lining around the epithelial nests was inapparent in the H&E section. Neither intracanalicular or pericanalicular growth pattern, nor stromal cell proliferation was found. The initial pathological diagnosis of the biopsy specimen based on the H&E section was invasive carcinoma, NOS. Total rather than partial mastectomy was performed to prevent her from applying post-operative radiation therapy because of her systemic scleroderma. The mammary tissue resected from the left upper lateral region included a well-bordered mass measuring approximately 12 x 9 mm, and the nodular lesion had similar histological findings of H&E sections to those of the biopsy specimen. There was only partial detection of intracanalicular structure (Figure 2a, 2b). Two specialists in breast pathology were consulted for the diagnosis of the tumor. One diagnosed FA without malignant potential, and the other diagnosed FA with ADH/DCIS. Following the advice of the pathologists, we performed immunohistochemical analysis by Ventana Benchmark ULTRA System (Roche Diagnostics, Basel, Switzerland). In immunohistochemical staining, tumor cells were diffusely positive for ER (Clone SP1, Roche Diagnostics) (Figure 2c) and completely negative for cytokeratin 5 (CK5) (Clone SP27, Roche Diagnostics) (Figure 2d). Also, CK5 highlighted myoepithelial lining around the tumor cell nests. E-cadherin (Clone 36, Roche Diagnostics) was positive at the tumor cell membrane. Our final diagnosis of the lesion was sclerosing FA with ADH/DCIS based on immunohistochemical results and the opinions of two specialists. The details of this case are reported with the patient’s permission.

## Discussion

There were arguments on the pathological diagnosis of the mammary lesion including the principal diagnosis and the malignant potential. The lesion had characteristic pathological findings including a small and well-bordered nodular mass, trabecular epithelial growth, and sclerotic stroma without stromal cell proliferation in both biopsy and resected specimens. In the resected specimens, intracanalicular structure was only partially found. Based on the histological findings of the well-defined border and partial intracanalicular structure of the lesion, we considered FA as the principal diagnosis of the lesion. FA sometimes has hyalinized hypocellular stroma, known as sclerotic FA, but sclerotic FA usually shows less growth of the epithelial component [2]. Sclerotic stroma of the lesion can be associated with scleroderma. Also, FA rarely shows epithelial proliferation revealing epithelial hyperplasia, microglandular adenosis, or sclerosing adenosis [2,3]. Usually, FA with sclerosing adenosis has various histological features, including cystic change, calcifications, and papillary apocrine metaplasia, as shown in complex FA [4]. To the extent of our knowledge, there have been no previous reports of FA with both trabecular epithelial proliferation of monotonous epithelial cells and totally hyalinized stroma.

The malignant potential in the lesion is a second issue in the pathological diagnosis. ADH, DCIS, and lobular carcinoma in situ may arise in FA [2-9]. Initial biopsy diagnosis of invasive carcinoma, NOS was unquestionably an overdiagnosis. This was due to the lack of distinct myoepithelial cells on the H&E section, but the myoepithelial cells were highlighted by CK5 immunohistochemical staining. On the other hand, monotonous epithelial growth of the lesion still suggests the potential for intraductal malignancy [7]. There was in fact discrepancy in diagnoses by the two pathology specialists regarding malignant potential in the FA. One of the specialists diagnosed FA with ADH/DCIS and the immunohistochemical results of diffuse positivity of ER and complete negativity of CK5 in monotonous epithelial cells suggested the possibility of ADH/DCIS. Based on the immunohistochemical results and low nuclear atypia of the epithelium, however, we made a final diagnosis of FA with low malignant potential [10]. Distinguishing between ADH and DCIS is difficult in such lesions, because we hardly estimate the volume of atypical epithelial proliferation in the lesion. Lobular carcinoma in situ, which may be in another differential diagnosis, was excluded because of the positivity of E-cadherin. Kuijper et al. reported that only 2% of patients with FA had malignancy and they suggested that the risk of progression to malignant potential in FA is extremely low [11]. Moreover, Carter et al. indicated that ADH in FA did not incur a significant risk of development to invasive carcinoma by a large cohort study of women with FA [12].

## Conclusions

In conclusion, FA rarely shows a dominant trabecular structure with epithelial atypia and sclerotic stroma, but this microscopic finding can mimic invasive carcinoma, NOS. Awareness of the unique histological subtype of FA is important in the diagnosis of biopsy specimens to avoid pathological misdiagnosis and clinical overtreatment. We report this case to promote consideration of our diagnosis of the biopsy specimen.
